# Multimorbidity and Peak Oxygen Uptake in Patients Undergoing Cardiac Rehabilitation

**DOI:** 10.1001/jamanetworkopen.2024.61331

**Published:** 2025-02-17

**Authors:** Pauline F. Gomes, Audry Chacin-Suarez, Sophie A. Miller, Karen M. Fischer, Thomas P. Olson

**Affiliations:** 1 Department of Cardiovascular Medicine, Mayo Clinic, Rochester, Minnesota; 2 Department of Medicine, Jefferson Einstein Philadelphia Hospital, Philadelphia, Pennsylvania; 3 Sidney Kimmel Medical College, Thomas Jefferson University, Philadelphia, Pennsylvania

## Abstract

This cohort study examines changes in oxygen consumption among patients with multiple morbidities undergoing cardiac rehabilitation.

## Introduction

The US Department of Health and Human Services defines multimorbidity as the simultaneous occurrence of 2 or more chronic conditions (CCs) requiring ongoing medical management for at least 1 year and contributing to functional limitations.^1^ The Centers for Medicare and Medicaid Services identified 21 CCs that impose increased burden on the nation’s health, including cardiovascular diseases and key risk factors. These conditions often coexist, creating complexity in formulating treatments and resulting in worse outcomes^2^

Cardiac rehabilitation (CR) is a comprehensive, secondary prevention program to help manage cardiovascular risk factors and improve cardiorespiratory fitness (CRF).^3^ Measurement of peak oxygen consumption (V̇o_2_peak) during cardiopulmonary exercise testing (CPET) is the criterion standard CRF metric.^4^ However, the effect of multimorbidity on improvements in V̇o_2_peak with CR remains underexplored.

## Methods

This cohort study was approved by Mayo Clinic’s institutional review board, and all participants provided authorization to use medical records for research purposes. Reporting adhered to the Strengthening the Reporting of Observational Studies in Epidemiology (STROBE) reporting guideline. We used the Rochester Epidemiology Project records linkage system to study patients with CR at Mayo Clinic, Rochester, Minnesota. Inclusion criteria were age at least 18 years, consent to have medical records used for research, and completion of CPET before and after CR. The CR program provides up to 36 sessions of aerobic and resistance training over 12 to 18 weeks tailored to individual needs, with guidance on medication adherence, nutrition, mental health, and lifestyle and behavioral modifications. All CPETs were conducted on treadmill or cycle ergometer by clinical exercise physiologists, with cardiologist oversight. Multimorbidity was assessed using *International Classification of Diseases, Ninth Revision *(*ICD-9*) codes. Multimorbidity was categorized using the median number of CCs (low, <6; high, ≥6). V̇o_2_peak improvement was calculated as percentage change before vs after CR, and we explored differences among improvement categories (≥1%, ≥5%, and ≥10%). Categorical variables were analyzed using χ^2^ test and noncategorical variables using Kruskal-Wallis test. *P* values were 2-sided, and statistical significance was set at *P* < .05. Logistic regression models were adjusted for age, sex, and baseline V̇o_2_peak to assess association between multimorbidity and change in V̇o_2_peak across categories. Data were analyzed using SAS software version 9.4 (SAS Institute) from August to November 2023.

## Results

The study included 620 participants (468 [75.5%] male; mean [SD] age, 62.9 [11.2] years) with mean (SD) body mass index (BMI; calculated as weight in kilograms divided by height in meters squared) 29.9 (5.4) and a median (range) of 6 (2-13) CC. Baseline mean (SD) V̇o_2_peak was 20.7 (6.3) mL/kg/min. Participants completed a mean (SD) of 28.9 (12.6) CR sessions. The most prevalent CCs were hyperlipidemia (96.1%), hypertension (78.4%), and cardiac arrhythmias (74.5%) (Table 1). Participants in the low CCs group were younger (mean [SD] age, 59.8 [11.2] vs 65.9 [10.2] years; *P* < .001) with higher baseline V̇o_2_peak (mean [SD], 23.0 [6.4] vs 18.5 [5.5] mL/kg/min; *P* < .001) compared with participants in the high CCs group (Table 1). Participants had a mean (SD) V̇o_2_peak change of 11.5% (21.7%), with 299 participants (48.2%) showing more than 10% improvement (Table 1). V̇o_2_peak improvement was higher in the low CCs group compared with the high CCs group (mean [SD] improvement, 12.5% [19.4%] vs 10.5% [23.7%]; *P* = .01). One-third of participants in the high CCs group (32.7%) did not demonstrate at least 1% improvement in V̇o_2_peak, compared with 23.6% of participants in the low CC group. Adjusted logistic regression showed participants with low CCs burden were more likely to improve V̇o_2_peak across all subcategories (ie, ≥1%, ≥5% and ≥10%) compared with those with high CCs (Table 2).

**Table 1.** Comparison of Demographic Characteristics and Change in V̇o_2_peak Between Groups With Multimorbidity According to Low and High Burden of CCs

Variables	Participants, No. (%)			*P* value
	CCs		Total (N = 620)	
	Low (<6) (n = 305)	High (≥6) (n = 315)		
Age, y				
Mean (SD)	59.8 (11.24)	65.9 (10.23)	62.9 (11.16)	<.001^a^
Median (IQR)	59.7 (53.2-67.2)	66.8 (59.2-73.7)	63.8 (55.1-71.6)	
Sex				
Male	256 (83.9)	212 (67.3)	468 (75.5)	<.001^b^
Female	49 (16.1)	103 (32.7)	152 (24.5)	
Race and ethnicity^c^				
American Indian or Alaskan Native	1 (0.3)	0	1 (0.2)	.15^b^
Asian	7 (2.3)	3 (1.0)	10 (1.6)	
Black or African American	3 (1.0)	3 (1.0)	6 (1.0)	
White	289 (94.8)	309 (98.1)	598 (96.5)	
Other	3 (1.0)	0	3 (0.5)	
Unknown	2 (0.7)	0	2 (0.3)	
BMI				
Mean (SD)	29.5 (5.35)	30.3 (5.47)	29.9 (5.42)	.02^a^
Median (IQR)	28.7 (25.8-31.8)	29.8 (26.8-33.5)	29.3 (26.1-33.0)	
CR sessions, No.				
Mean (SD)	27.8 (13.33)	29.9 (11.70)	28.9 (12.56)	.05^a^
Median (IQR)	33.0 (19.0-35.0)	33.0 (24.0-35.0)	33.0 (22.0-35.0)	
Baseline V̇o_2_peak, mL/kg/min				
Mean (SD)	23.0 (6.38)	18.5 (5.45)	20.7 (6.33)	<.001^a^
Median (IQR)	22.1 (18.3-26.9)	18.2 (14.2-22.1)	20.0 (16.5-24.3)	
Follow-up V̇o_2_peak, mL/kg/min				
Mean (SD)	25.5 (7.23)	20.0 (6.13)	22.7 (7.23)	<.001^a^
Median (IQR)	25.3 (20.5-30.3)	19.6 (15.6-24.2)	21.8 (17.6-26.8)	
Change in V̇o_2_peak, %				
Mean (SD)	12.5 (19.35)	10.5 (23.69)	11.5 (21.67)	.01^a^
Median (IQR)	11.6 (1.4-21.7)	7.6 (−2.4-19.5)	9.0 (0.0-20.6)	
1% Change V̇o_2_peak				
Below	72 (23.6)	103 (32.7)	175 (28.2)	.01^b^
Above	233 (76.4)	212 (67.3)	445 (71.8)	
5% Change V̇o_2_peak				
Below	106 (34.8)	135 (42.9)	241 (38.9)	.04^b^
Above	199 (65.2)	180 (57.1)	379 (61.1)	
10% Change V̇o_2_peak				
Below	145 (47.5)	176 (55.9)	321 (51.8)	.04^b^
Above	160 (52.5)	139 (44.1)	299 (48.2)	
CC				
Autism^d^	0	0	0	NA
Cancer	41 (13.4)	148 (47.0)	189 (30.5)	<.001^b^
Cardiac arrhythmias	182 (59.7)	280 (88.9)	462 (74.5)	<.001^b^
Chronic heart failure	34 (11.1)	97 (30.8)	131 (21.1)	<.001^b^
Chronic kidney disease	12 (3.9)	101 (32.1)	113 (18.2)	<.001^b^
Chronic pulmonary disease	46 (15.1)	158 (50.2)	204 (32.9)	<.001^b^
Coronary artery disease^d^	291 (95.4)	311 (98.7)	602 (97.1)	.01^b^
Dementia	1 (0.3)	22 (7.0)	23 (3.7)	<.001^b^
Depression	36 (11.8)	136 (43.2)	172 (27.7)	<.001^b^
Diabetes	96 (31.5)	231 (73.3)	327 (52.7)	<.001^b^
Hepatitis	8 (2.6)	13 (4.1)	21 (3.4)	.30^b^
HIV^d^	0	1 (0.3)	1 (0.2)	.32^b^
Hyperlipidemia	285 (93.4)	311 (98.7)	596 (96.1)	.001^b^
Hypertension	196 (64.3)	290 (92.1)	486 (78.4)	<.001^b^
Osteoporosis	7 (2.3)	41 (13.0)	48 (7.7)	<.001^b^
Schizophrenia	0	7 (2.2)	7 (1.1)	.009^b^
Stroke	18 (5.9)	87 (27.6)	105 (16.9)	<.001^b^
Substance abuse disorder	18 (5.9)	50 (15.9)	68 (11.0)	<.001^b^

Abbreviations: BMI, body mass index (calculated as weight in kilograms divided by height in meters squared); CC, chronic conditions; CR, cardiac rehabilitation; NA, not applicable; V̇o_2_peak, peak oxygen consumption.

^a^ Kruskal-Wallis *P* value.

^b^ χ^2^ *P* value.

^c^ Race and ethnicity were self-identified. Participants did not provide further information when identifying into the other race and ethnicity group.

^d^ Autism and HIV were excluded due to low prevalence. Coronary artery disease was excluded due to high prevalence.

**Table 2.** Estimated Association Between Burden of CCs and Change in Peak Oxygen Consumption From Logistic Regression Models^a^

Change	Model, low vs high CC burden			
	Unadjusted		Adjusted^b^	
	OR (95% CI)	*P* value	OR (95% CI)	*P* value
≥1%	1.57 (1.10-2.24)	.01	1.73 (1.17-2.57)	.006
≥5%	1.41 (1.02-1.95)	.04	1.58 (1.10-2.28)	.01
≥10%	1.40 (1.02-1.92)	.04	1.59 (1.11-2.28)	.01

Abbreviations: CC, chronic condition; OR, odds ratio.

^a^ Autism, coronary artery disease, and HIV were excluded from the total comorbidity score.

^b^ Adjusted for sex, age, and baseline oxygen consumption.

## Discussion

The findings of this cohort study suggest that although CR was associated with improved CRF, the burden of multimorbidity may impair the degree of improvement. This aligns with previous studies finding that CR is associated with improved CRF,^5^ while other studies have found a cycle of deconditioning and worsening health outcomes with greater numbers of CCs.^6^ This suggests a tailored strategy designed to address cardiac and noncardiac CCs in CR patients may be needed to optimize CRF improvements. However, this study has limitations, including single-center design, predominantly male sample, and retrospective design, which introduce potential biases. While these factors may affect generalizability, internal validity is preserved. Our study highlights the important negative associations of multimorbidity burden with CRF improvement in CR patients. Additional studies are needed to evaluate tailored CR programming approaches specifically targeting patients with high multimorbidity burden.

## Notes

Supplement.
Data Sharing Statement
