# Supplementary material for: Multimorbidity and Peak Oxygen Uptake in Patients Undergoing Cardiac Rehabilitation
Source: JAMA Netw Open. 2025 Feb 17;8(2):e2461331. doi: 10.1001/jamanetworkopen.2024.61331 (PMC11833515; doi:10.1001/jamanetworkopen.2024.61331)
Supplement: Supplement. — Data Sharing Statement [file jamanetwopen-e2461331-s001.pdf]

Data Sharing Statement

Gomes. Multimorbidity and Peak Oxygen Uptake in Patients Undergoing Cardiac Rehabilitation. *JAMA Netw Open*. Published February 17, 2025.
doi:10.1001/jamanetworkopen.2024.61331

Data
Data available: No
